# Onychomycosis among Clinically Suspected Cases Attending the Dermatology Out-patient Department of a Tertiary Care Centre: A Descriptive Cross-sectional Study

**DOI:** 10.31729/jnma.6277

**Published:** 2021-05-31

**Authors:** Beena Jha, Manisha Sharma, Sagar GC, Jyotshna Sapkota

**Affiliations:** 1 Department of Microbiology, Kathmandu Medical College and Teaching Hospital, Kathmandu, Nepal; 2 Department of Dermatology and Venereology, Kathmandu Medical College and Teaching Hospital, Kathmandu, Nepal

**Keywords:** *dermatophytes*, *onychomycosis*, *trichophyton*

## Abstract

**Introduction:**

Onychomycosis is a fungal disease of the nail apparatus caused by dermatophytes, non-dermatophytes and yeasts. Although onychomycosis is rarely life threatening, its high incidence and the associated morbidity makes it an important public health problem. This study was aimed to find the prevalence of onychomycosis among clinically suspected patients attending the outpatient department of Dermatology and Venereology.

**Methods:**

A descriptive cross-sectional study was done in a total of 200 clinically suspected cases of onychomycosis attending the Dermatology outpatient department of a tertiary care hospital within the period of one year from 1st September 2019 to 31st August 2020. Ethical approval (Reference: 150320196) was taken and convenience sampling was done. Data were analyzed using Statistical Package for Social Sciences version 19. Point estimate at 95% Confidence Interval was calculated along with frequency and proportion for binary data.

**Results:**

Out of 200 clinically suspected cases prevalence of onychomycosis was found to be 40 (20%) (Confidence Interval = 19.94-20.06) by both direct microscopy and culture. Onychomycosis was commonest among age group of 21-40 years and more predominant among male patients 60 (65.21%). The fingernails were frequently involved i.e., 58 (63%) cases followed by toenails 34 (21%). Dermatophytes were the most common type of fungal infection accounting for 25 (62.5%).

**Conclusions:**

The study highlighted dermatophytes as the most common clinical pattern of onychomycosis and Trichophyton rubrum as the most common aetiological agent causing onychomycosis.

## INTRODUCTION

Onychomycosis is fungal infections of nail that causes discoloration, thickening, and separation from the nail bed.^1^ Onychomycosis is caused by dermatophytes, nondermatophyte moulds and yeasts. It is common infection in adults and accounts for 20% of all nail diseases.^2^ It affects approximately 5% of the population worldwide.^3^

Although onychomycosis is rarely life threatening, its high incidence, prevalence and the associated morbidity makes it an important public health problem.^4^ The prevalence of fungal nail infections worldwide ranges from 3-26%, about 3-8% prevalence has been reported in the United Kingdom and 2-3% in US.^4,5^

At present, clinicians rely on clinical examination and a combination of direct microscopy and fungal culture to achieve a diagnosis. Identification of the specific causative organism is important because some organisms are less likely to respond to certain antifungal agents. This study was conducted to know the prevalence of onychomycosis among the patients attending outpatient department of Dermatology and Venereology.

## METHODS

A descriptive cross-sectional study was conducted within the period of 1 year from 1st September 2019 to 31st August 2020, among the patients attending the outpatient Department of Dermatology and Venereology of Kathmandu Medical College and Teaching Hospital. Ethical clearance was obtained from Institutional Review Committee (Reference number: 150320196) before this study was conducted. Convenience sampling was done and the sample size was calculated from study done in Nepal with culture positive rate of 51.4%.^6^ The sample size was calculated using formula,


$$\begin{array}{ll} \text{n} & \text{= }{\text{Z}}^{\text{2}}\times \text{p}\times \text{q}/{\text{e}}^{\text{2}}\\ & \text{= }{\left(1.96\right)}^{\text{2}}\times (0.514)\times (0.486)/{(\text{0.07)}}^{\text{2}}\\ & \text{= }195.7\\ & =196 \end{array}$$

Where,

n = minimum required sample size
Z = 1.96 at 95% Confidence Interval (CI)
p = past prevalence, 51.4%^6^
q = 1-p
e = margin of error, 7%

The calculated sample size is 196 however sample size of 200 was taken. All infected nail samples were collected from 200 clinically suspected cases of onychomycosis. Analysis of the data was performed using Statistical Package for Social Sciences (SPSS) version 19.0. Point estimate at 95% CI was calculated along with frequency and proportion for binary data.

## RESULTS

Out of 200 clinically suspected cases prevalence of onychomycosis was found to be 40 (20%) at 95% Confidence Interval (19.94-20.06) by both direct microscopy and culture.

Among, 200 clinically suspected cases of onychomycosis, 92 (46%) were positive in direct microscopy, however only a total of 40 (20%) were positive by culture. Similarly, 52 (26%0 cases were positive by direct microscopy but negative by culture and 108 (54%) were negative both by direct microscopy and culture.

The highest incidence was found in age group of 21-40 years followed by 41-60 years. Infection rate was less in age group below 20 years. The fingernails were more frequently involved i.e., 58 (63%) cases followed by toenails 34 (21%). Ratio of fingernail to toenail infection was 3:1 as shown in (Table 1).

**Table 1:** Age wise distribution of onychomycosis in relation with site involved.

Age group	<20 n (%)	21-40 n (%)	41-60 n (%)	>60 years n (%)
Finger nails	6 (6.52)	24 (26.08)	18 (19.56)	10 (10.86)
Toe nails	4 (4.3)	10 (10.86)	12 (13.04)	8 (8.69)

Out of 92 KOH positive cases, male were commonly infected 60 (65.21%) whereas 32 (34.78%) were only female patients as shown in (Table 2). Male to female ratio in our study was 1.8:1.

**Table 2:** Gender wise distribution of onychomycosis among KOH positive.

Gender	n (%)
Male	60 (65.21)
Female	32 (34.78)

Culture and KOH positive were 40 (20%) while culture and KOH negative were 108 (54) as shown in (Table 3).

**Table 3:** Findings of KOH and culture examination.

	KOH + ve n (%)	KOH-ve	Total n (%)
Culture positive	40 (20)	0 (0)	40 (20)
Culture negative	52 (26)	108 (54)	160 (80)
Total	92 (46)	108 (54)	200 (100)

Distal and lateral subungual onychomycosis (DLSO) was the commonest clinical pattern 92 (46%) followed by proximal subungual onychomycosis 43 (21.5%) and then total dystrophic onychomycosis 45 (22.5%), and SWO 20 (10%) as shown in (Table 4).

**Table 4:** Distribution of superficial fungal infection by clinical type.

Type of lesion	n (%)
Disto- Lateral subungual onychomycosis	92 (46)
Proximal subungual onychomycosis	43 (21.5)
Superficial white onychomycosis	20 (10)
Total dystrophic onychomycosis	45 (22.5)

Among the microscopy and culture positive cases dermatophytes were the most common type of fungal infection accounting for 25 (62.5%) followed by nondermatophytes 14 (35%) and candida by 1 (2.5%) (Table 5).

**Table 5:** Culture results of onychomycosis.

Organism	n (%)
Dermatophytes	25 (62.5)
Trichophyton rubrum	10 (25)
T. mentagrophytes	8 (20)
T. tonsurans	7 (17.5)
Nondermatophytes	14 (35)
Alternaria spp.	3 (7.5)
Cladosporidiumspp.	3 (7.5)
Fusarium spp.	3 (7.5)
Sepedonium spp.	2 (5)
Aspergillus niger	1 (2.5)
A. flavus	1 (2.5)
Rhizopus spp.	1 (2.5)
Candida spp.	1 (2.5)

## DISCUSSION

Onychomycosis is a common infection of nails in adults and accounts for 2 to 50% worldwide and the incidence increases with age.^7^ The prevalence of fungal nail infection in this study was 20% which correlates with other studies. The disease can occur at any age, but is more common between 40 to 60 years of age.^8-10^ In contrast to this commonly affected age group, the majority of our patients were between 21 to 40 years followed by 41 to 60. This is in accordance with the reports of various studies done in Nepal and India.^6,11,13^ Infections were less common in the age group below 20 years.

Predisposing factors for onychomycosis include diabetes mellitus, older age, hyperhidrosis, onychogryphosis, nail trauma, poor peripheral circulation, and immunosuppression.^15^ Higher incidence was noted amongst males (65%) than females, the ratio being 1.8:1, which compares well with most of the studies done in India by Veer P, et al. and Agarwalla A, et al. in Nepal,^6,11-14^ Higher incidence in males may be because they are more exposed to outdoors with greater physical activity and are more prone to trauma and longer use of occlusive footwear in males compared to females. High incidence of onychomycosis of the toenails have been reported by Gupta, et al.^9^ In the present study there were more cases of fingernail onychomycosis, than toenails, which compares well with other studies.^6,11,13^ Incidence of increased finger nail onychomycosis may be because of the increased chances of occupation related trauma, also fingernail infection is more likely than the toenail infection to arouse the patients concern, driving them to seek medical attention. The high incidence of DLSO pattern has been reported by various studies. Incidence of DLSO in present study was about 46% which is comparable with the study done by Garg, et al. 64.4%.^12^ Relative lack of effective cell-mediated immunity in the nail apparatus seems to make the nail more vulnerable to fungal infections.^15^ Once fungus is established in nails, infected nails act as a reservoir of an organism providing a constant source of infection for other parts of the body as suggested by the presence of concurrent fungal infection. In the present study, dermatophytes were the commonest isolates accounting 62.5% of all cultures, which is in accordance with the study done in India by Veer, et al.^12^ T. rubrum was the common isolate i. e., 65% which is similar with other studies.^2,5^ On contrary to our study nondermatophytes were the commonest isolates in a study conducted in India by Raghbendra KR in India.^16^ There was an increasing trend of NDM 35% observed in our study which is comparable with Grover, et al.^5^ Candida was the most common isolates accounting 46.0% followed by dermatophytes (43.0%) and non-dermatophyte molds (NDM 11.0%) in a study done by Bokhari, et al.^10^ Current research techniques of molecular biology such as dual flow cytometry have produced new and convincing evidence to differentiate fungal species and for quantitating the fungal pathogen in nails.^17^ These procedures are not available in Nepal.

## CONCLUSIONS

The present study concludes that the prevalence of onychomychosis was low. T. rubrum and T. mentagrophytes are the major pathogens of onychomycosis in central Nepal. There is need to develop rapid diagnostic tests for onychomycosis to reduce the practice of empirical treatment. This study also emphasizes the role of NDM associated onychomycosis.
